# Coated sodium butyrate ameliorates high-energy and low-protein diet induced hepatic dysfunction via modulating mitochondrial dynamics, autophagy and apoptosis in laying hens

**DOI:** 10.1186/s40104-023-00980-8

**Published:** 2024-02-02

**Authors:** Sasa Miao, Tianming Mu, Ru Li, Yan Li, Wenyan Zhao, Jiankui Li, Xinyang Dong, Xiaoting Zou

**Affiliations:** Key Laboratory of Animal Feed and Nutrition of Zhejiang Province, Key Laboratory of Animal Nutrition and Feed Science (Eastern of China), Ministry of Agriculture and Rural Affairs, The Key Laboratory of Molecular Animal Nutrition, Ministry of Education, College of Animal Sciences, Zhejiang University, Hangzhou, 310058 China

**Keywords:** Autophagy, Coated sodium butyrate, Laying hens, Lipid metabolism, Mitochondria

## Abstract

**Background:**

Fatty liver hemorrhagic syndrome (FLHS), a fatty liver disease in laying hens, poses a grave threat to the layer industry, stemming from its ability to trigger an alarming plummet in egg production and usher in acute mortality among laying hens. Increasing evidence suggests that the onset and progression of fatty liver was closely related to mitochondria dysfunction. Sodium butyrate was demonstrated to modulate hepatic lipid metabolism, alleviate oxidative stress and improve mitochondrial dysfunction in vitro and mice models. Nevertheless, there is limited existing research on coated sodium butyrate (CSB) to prevent FLHS in laying hens, and whether and how CSB exerts the anti-FLHS effect still needs to be explored. In this experiment, the FLHS model was induced by administering a high-energy low-protein (HELP) diet in laying hens. The objective was to investigate the effects of CSB on alleviating FLHS with a focus on the role of CSB in modulating mitochondrial function.

**Methods:**

A total of 288 healthy 28-week-old Huafeng laying hens were arbitrarily allocated into 4 groups with 6 replicates each, namely, the CON group (normal diet), HELP group (HELP diet), CH500 group (500 mg/kg CSB added to HELP diet) and CH750 group (750 mg/kg CSB added to HELP diet). The duration of the trial encompassed a period of 10 weeks.

**Results:**

The result revealed that CSB ameliorated the HELP-induced FLHS by improving hepatic steatosis and pathological damage, reducing the gene levels of fatty acid synthesis, and promoting the mRNA levels of key enzymes of fatty acid catabolism. CSB reduced oxidative stress induced by the HELP diet, upregulated the activity of GSH-Px and SOD, and decreased the content of MDA and ROS. CSB also mitigated the HELP diet-induced inflammatory response by blocking *TNF-α, IL-1β*, and F4/80. In addition, dietary CSB supplementation attenuated HELP-induced activation of the mitochondrial unfolded protein response (UPRmt), mitochondrial damage, and decline of ATPase activity. HELP diet decreased the autophagosome formation, and downregulated LC3B but upregulated p62 protein expression, which CSB administration reversed. CSB reduced HELP-induced apoptosis, as indicated by decreases in the *Bax*/*Bcl-2*, *Caspase-9*, *Caspase-3,* and *Cyt C* expression levels.

**Conclusions:**

Dietary CSB could ameliorate HELP diet-induced hepatic dysfunction via modulating mitochondrial dynamics, autophagy, and apoptosis in laying hens. Consequently, CSB, as a feed additive, exhibited the capacity to prevent FLHS by modulating autophagy and lipid metabolism.

**Graphical Abstract:**

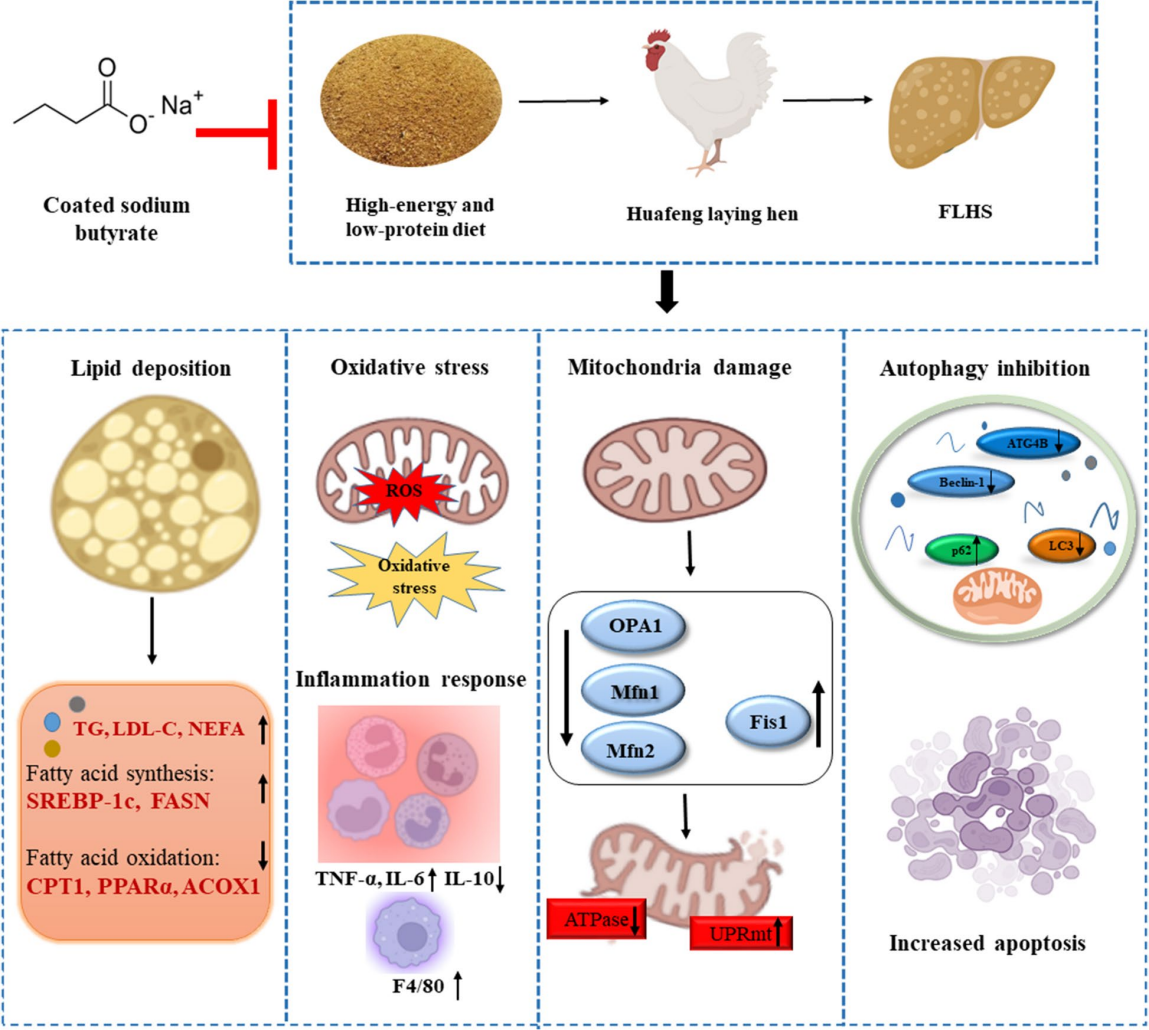

**Supplementary Information:**

The online version contains supplementary material available at 10.1186/s40104-023-00980-8.

## Background

Fatty liver hemorrhagic syndrome (FLHS), a fatty liver disease affecting avian species, is characterized by elevated triglyceride in hepatocytes accompanied by hepatic steatosis, hepatomegaly, and hemorrhage [1]. As the most common non-communicable disease, FLHS has emerged as a prominent cause of mortality in commercial layers hens [2], resulting in substantial economic ramifications for the poultry industry [3]. Existing literature reports that, within conventional poultry farming, FLHS contributes to mortality rates ranging from 28% to 74% [3, 4]. The etiology of FLHS is multifactorial, encompassing considerations such as nutrition, environment, genetics, and medication usage [4–6]. Nonetheless, excessive caloric intake emerges as the predominant driving factor, as FLHS can be induced by a high-energy diet [7–9]. The pathological and physiological mechanisms underlying FLHS remain unclear, but it shares similarities with non-alcoholic fatty liver disease (NAFLD), relating to hepatic inflammatory response, oxidative stress, autophagy, and apoptosis [8, 10, 11].

Unlike mammals, 90%–95% of the new fatty acid production occurs in the liver in avian species [12]. Lipid catabolism in the liver mightily rely on mitochondrial metabolic functions [13]. Mitochondria, in addition to their role as the primary energy-providing organelles, actively contribute to the generation of reactive oxygen species (ROS), which are typically counterbalanced by antioxidants within the mitochondria [14]. Within the hepatic cells afflicted with NAFLD, an imbalance ensues, characterized by the excessive accumulation of ROS and a concomitant reduction in mitochondrial antioxidant capacity, leading to a surge in lipid peroxidation and subsequent mitochondrial impairment [15]. In turn, mitochondrial impairment leads to the dysregulation of mitochondrial fusion and fission processes, resulting in perturbed mitochondrial morphology, compromised oxidative metabolism, and cell apoptosis and ultimately culminating in lipid deposition and liver damage [16]. To combat these challenges, the organism employs defense mechanisms such as mitochondrial biogenesis, unfolded protein response in mitochondria (UPRmt), and autophagy to maintain the mitochondrial quality during stress conditions, thereby ensuring the relative stability of mitochondria [16, 17]. Autophagy is an evolutionarily conserved cellular process that involves the degradation of damaged cytoplasmic components, such as organelles and protein aggregates via the lysosomal apparatus, followed by subsequent reuse [18]. Mitophagy is a selective autophagy process, primarily responsible for the degradation of damaged or excess mitochondria via lysosomes [19, 20]. Additionally, extensive research has demonstrated the involvement of autophagy in regulating cellular lipid homeostasis [21, 22]. Autophagy activation exerts a lipid-lowering effect through the modulation of lipid metabolism protein expression, while autophagy deficiency exacerbates lipid deposition and impairs mitochondrial function [23]. Furthermore, restoration of autophagic flux has the potential to attenuate or impede the progression of fatty liver [24]. Therefore, dietary interventions with bioactive substances that are able to mitigate hepatic mitochondrial damage and maintain mitochondrial homeostasis are potential therapeutic strategies for FLHS.

Butyric acid, produced by gut microbes from indigestible foods [25], has the potential to mitigate the risk of metabolic syndrome. Butyric acid possesses anti-inflammatory and anti-oxidative properties and has been demonstrated to alleviate hepatic steatosis and dyslipidemia in the cell, rat and mouse models [26–30]. Recent research highlighted its protective effects against obesity, hepatic steatosis, and insulin resistance in mice fed a high-fat diet [31]. The mechanism of action of butyrate involved the increase of energy expenditure and mitochondrial function in adipose tissue and muscle in mice [32]. Mollica et al. [33] have also shown that sodium butyrate administration to obese mice with insulin resistance can reduce ROS production and increase hepatic mitochondrial function. Our recent study has indicated that coated sodium butyrate (CSB) could ameliorate hepatic lipid accumulation and inflammation in post-peaking laying hens [34]. Nevertheless, limited research exists on CSB administration to prevent FLHS from laying hens, especially focusing on a mitochondrial perspective in laying hens. Therefore, we hypothesize that CSB could alleviate FLHS caused by the high-energy low-protein (HELP) diet by mitigating lipid accumulation and improving mitochondria-related functions. This study aims to explore the protective effects of CSB on FLHS induced by the HELP diet, contributing to the theoretical foundation for CSB's potential application in FLHS prevention.

## Methods

### Experimental design, animals, and diet

Following a one-week acclimatization period, 288 healthy 28-week-old Huafeng laying hens were randomly divided into 4 groups, each with 6 replicates, namely, the CON group (normal diet), HELP group (high-energy and low-protein diet), CH500 group (HELP diet with 500 mg/kg CSB) and CH750 group (HELP diet with 750 mg/kg CSB). The laying period of Huafeng laying hens ranges from around 140 to 500 days of age. The CSB, with 50% sodium butyrate content coated with silica and palm oil, was sourced from Hangzhou Dade Biotechnology Co., Ltd. (Hangzhou, China). The section on CSB dosage in this experiment was guided by our previous report [34], which had a positive effect by mitigating hepatic lipid accumulation and inflammatory responses in Huafeng laying hens. The duration of the trial encompassed a period of 10 weeks, during which hens had unrestricted access to feed and water. The coop underwent disinfection regularly, while ventilation and lighting conditions remained consistent, maintaining an average daily light exposure of 16 h. The CON and HELP group diet composition during the study period are displayed in Table 1.

**Table 1:** Ingredient compositions and nutrient levels of the diet

Item	Normal diet	High-energy low-protein diet
Ingredient composition, %		
Corn	64.50	69.70
Soybean meal	24.00	14.58
Corn oil	0	4.22
Limestone	8.00	8.00
CaHPO_4_	1.20	1.20
NaCl	0.30	0.30
Premix^a^	2.00	2.00
Total	100.00	100.00
Nutrient level (air-dry basis)		
Crude protein, %	15.86	12.00
Calcium, %	3.43	3.61
Available phosphorus, %	0.51	0.46
Arginine, %	1.03	0.74
Methionine, %	0.38	0.36
Lysine, %	0.93	0.69
Valine, %	0.77	0.58
Methionine + Cysteine, %	0.67	0.56
Energy, kcal/kg	2,678.99	3,100.00

^a^Per kilogram of additives contained the following: Cu, 2.50 mg; Fe, 20.00 mg; Zn, 17.50 mg; Mn, 15.00 mg; KI, 4.00 mg; Na_2_SeO_3_, 6.00 mg; CoCl_2_·6H_2_O, 2.50 mg; Met, 50.00 mg; chromium, 2.00 mg; phytase, 10.00 mg; kininase, 7.50 mg; antioxidant, 2.00 mg; betaine, 15.00 mg; choline, 50.00 mg; NaCl, 200.00 mg; Ca-P, 500.00 mg; zeolite, 76.00 mg

### Sample collection

At the end of the trial, two hens per replicate were randomly chosen for a 12-h fasting period. Blood samples were collected from wing veins into the tubes containing a pro-coagulant, and then centrifuged at 3,000 r/min for 10 min to isolate serum. The obtained serum was immediately preserved at −80 °C for subsequent analysis. Afterwards, the hens were slaughtered, and a portion of fresh liver from the left lobe was taken for fixation. The remaining left lobe liver tissue was divided into thirds and promptly collected, then preserved at −80 °C for subsequent detection.

### Serum biochemical assessments

The activities or contents of total cholesterol (TC), triglyceride (TG), low-density lipoprotein cholesterol (LDL-C), high-density lipoprotein cholesterol (HDL-C), aspartate transferase (AST), and alanine aminotransferase (ALT), nonesterified fatty acids (NEFA) were monitored using the microplate method according to the respective kit instruction manuals (A111-1-1, A110-1-1, A113-1-1, A112-1-1, C010-2-1, C009-2-1 and A042-2-1, Nanjing Jiancheng Bioengineering Institute, Nanjing, China).

### Liver parameters detection

Liver tissue samples weighing approximately 0.5 g from part of the left lobe (*n* = 6 layers each group) were subjected to homogenization in 4.5 mL of phosphate buffer saline. The samples were then centrifuged at 3,000 r/min for 10 min at 4 °C. The resulting supernatant was collected, yielding a 10% liver homogenate. Liver homogenates were utilized to measure the activity or content of antioxidant substrates, including glutathione peroxidase (GSH-Px), superoxide dismutase (SOD), catalase (CAT), malondialdehyde (MDA) as the operation manual described (A005-1, A001-3, A007-1-1 and A003-1, Nanjing Jiancheng Bioengineering Institute, Nanjing, China). GSH-Px activities were assessed by colorimetric assay, while SOD, CAT, and MDA activities were determined using by microplate assay.

The 10% liver homogenate was centrifuged at 2,000 r/min for 10 min. Subsequently, the supernatant was isolated by further centrifugation at 10,000 r/min for 15 min to obtain precipitate, which is liver mitochondria. ATPase activities, including Ca^2+^Mg^2+^-ATPase, Mg^2+^-ATPase, Ca^2+^-ATPase, and Na^+^K^+^-ATPase, were assayed colorimetrically on the obtained liver mitochondria using manufacturer's instructions (A016-2, Nanjing Jiancheng Bioengineering Institute, Nanjing, China).

### Histological analysis

After the experiment, fresh liver tissue specimens from part of the left lobe measuring approximately 0.2 cm × 0.2 cm were fixed using a 4% paraformaldehyde solution. The fixed liver samples were embedded in paraffin and then transversely sectioned into 5 µm slices. Following standard histopathological procedures, liver sections were stained with hematoxylin–eosin (H&E). Additionally, the fixed liver tissues were embedded using the optimum cutting temperature compound for the frozen sections (5 µm thick), which were stained with Oil Red O. An optical microscope (Nikon Eclipse Ni-U, Tokyo, Japan) was used to examine the sections and capture images.

### Liver ultrastructural observation

After the experiment, fresh liver tissue blocks, measuring 1 mm × 1 mm × 1 mm, were excised from part of the left lobe and promptly immersed in a 2.5% (v/v) glutaraldehyde solution for fixation. After rinsing phosphate-buffered saline, the specimens were further immersed in 1% osmium tetroxide for 1 h. Following ethanol dehydration and embedding, thin sections were cut at a thickness of 70 nm and mounted on copper grids. Then, these sections underwent staining with Mg-uranyl acetate and Pb-citrate to facilitate observation under transmission electron microscopy (GEM-1200ES, Japan Electron Optics Laboratory Co., Ltd., Tokyo, Japan).

### Immunofluorescence (IF) staining detection and TUNEL assay

Fresh liver tissues from part of the left lobe were immediately sampled and stored at −20 °C, after which they were made into 10 µm frozen sections for ROS content using dihydroethidium (DHE) staining. Additionally, the paraffin-embedded liver sections, made as above, underwent a dehydration process using ethanol solutions and were rendered transparent using xylene. The sample slides were blocked with 1% BSA for 1 h, followed by incubation with F4/80 (dilution 1:500, GB113373, Servicebio, Wuhan, China) or LC3B (dilution 1:200, AF300985, Hunan Aifang Biotechnology, Changsha, China), or p62 (dilution 1:500, GB11531, Servicebio, Wuhan, China) antibodies at 4 °C overnight. After that, the sections were incubated with a secondary antibody and counterstained with 4,6-diamidino-2-phenylindole (DAPI, G1012, Servicebio, Wuhan, China).

The TUNEL assay was conducted using the one-step TUNEL apoptosis assay kit (C1088, Beyotime Biotechnology, Shanghai, China); Briefly, paraffin-embedded liver sections were deparaffinized, rehydrated, and incubated with digestive proteinase K for antigen extraction at 37 °C for 20 min. Then, they were incubated with 5 μL TdT enzyme and 45 μL fluorescein-dUTP at 37 °C for 1 h. After washing, slides were stained with DAPI to visualize the nuclei. Images of all the above-processed slides were captured with a confocal microscope (FV 1000; Olympus, Tokyo, Japan), and fluorescence intensity was quantified using Image J software.

### Quantitative real-time PCR

Approximately 50 mg of liver tissue from part of the left lobe was used for total RNA extraction, employing 1 mL TRIzol reagent, followed by reverse transcription in accordance with the cDNA synthesis kit instructions (R223, Vazyme, Nanjing, China). Quantitative real-time PCR (qRT-PCR) was conducted using the SYBR Green Master Mix (Q711, Vazyme, Nanjing, China) on the Applied Biosystems QuantStudio 3 Real-Time PCR System. To ensure reliability, the Ct value for *β-actin* was consistently below 0.5 across all tissue types, establishing it as a suitable endogenous control. The relative mRNA expression of target genes was normalized to that of *β-actin* levels using the comparative 2^−△△Ct^ [35]. The primer sequences employed for qRT-PCR are detailed in Table S1.

### Statistical analysis

The data were analyzed using one-way ANOVA in SPSS 20.0 software, followed by post hoc multiple comparisons with Tukey's test. Graphs were generated using GraphPad Prism 8.0 software. Results are presented as mean ± SEM. Furthermore, the clustering-related heat maps and networks with flags and principal component analysis (PCA) were constructed using the Tutools platform (http://cloudtutu.com.cn).

## Results

### CSB improved liver morphology and function in laying hens fed the HELP diet

**Fig. 1:**
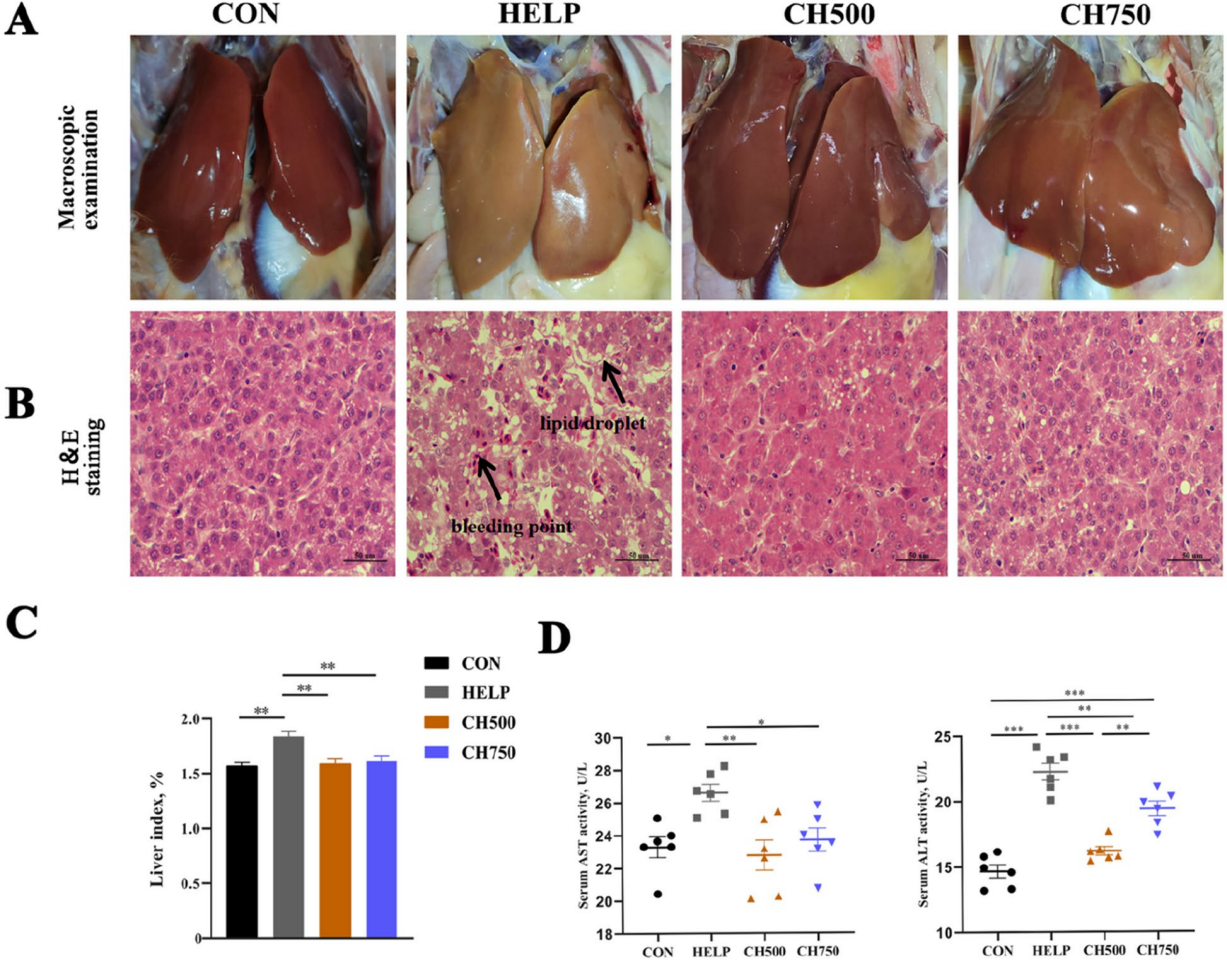
CSB improved liver morphology and function in laying hens fed the HELP diet. **A** Representative images of liver dissection (*n* = 6). **B** Representative photomicrographs of H&E staining (*n* = 6). **C** Liver index = liver weight/body weight × 100 (*n* = 6). **D** Serum AST and ALT activity levels (*n* = 6). Data are presented as mean ± SEM. ^*^P < 0.05, ^**^P < 0.01, ^***^P < 0.001

As illustrated in Fig. 1A, the hepatic macroscopic alterations in birds from the HELP group turned visibly yellow as well as enlarged and hemorrhagic compared with the CON group. Nevertheless, the alterations were notably alleviated by the addition of CSB, as observed in the CH500 and CH750 groups. This trend is also reflected in the liver index results (Fig. 1C). As showed by H&E staining (Fig. 1B), the hepatic histology in the HELP group displayed multiple vesicular steatoses accompanied by hemorrhagic spots compared with the CON group. Nevertheless, the deterioration caused by the HELP diet was distinctly attenuated by administration with CSB. Subsequently, we tested serum transaminase activities (Fig. 1D). Results displayed that the HELP diet caused an obvious rise in serum AST and ALT activities compared to CON diet, whereas CSB administration inhibited their rise.

### CSB alleviated HELP diet-induced oxidative stress

**Fig. 2:**
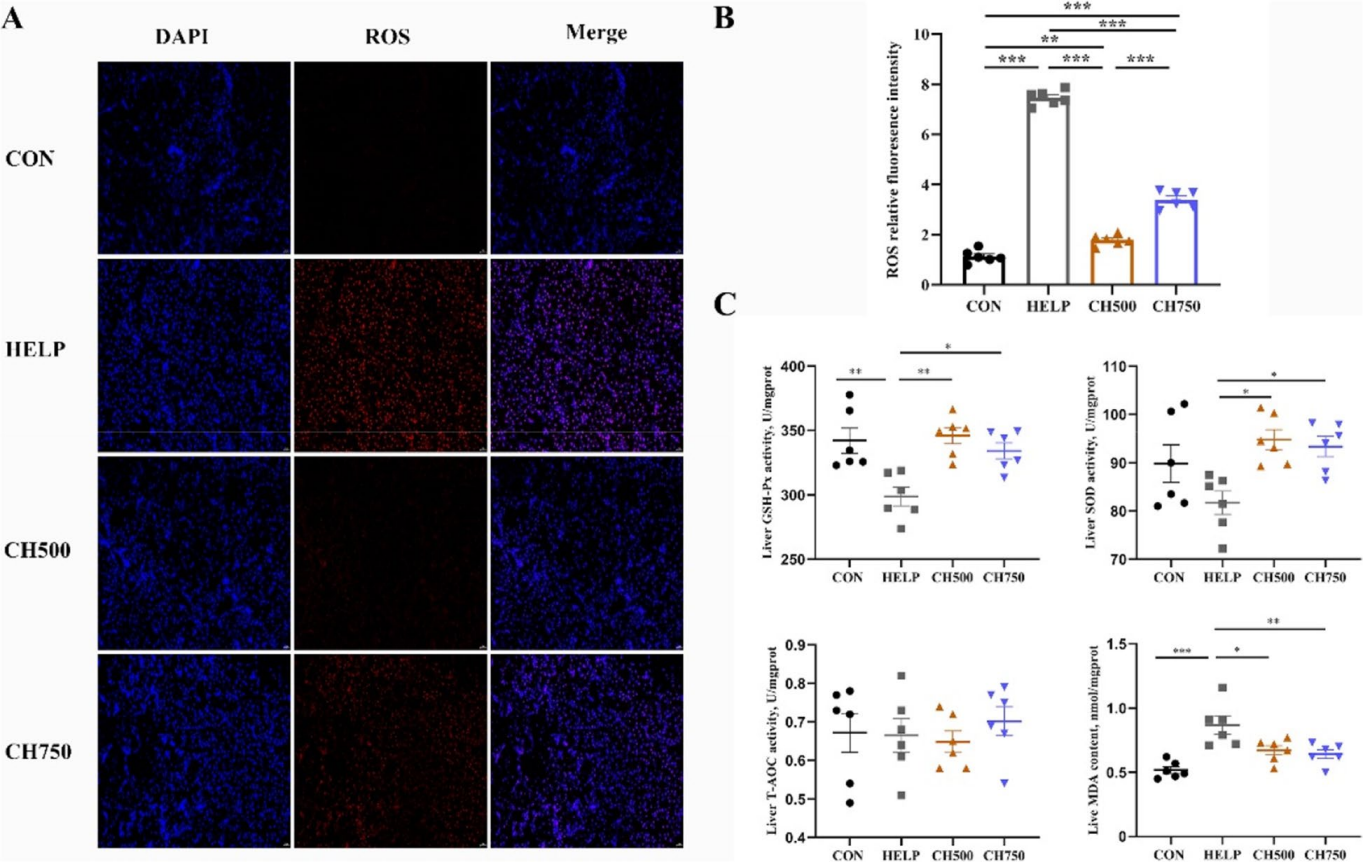
CSB alleviated HELP diet-induced oxidative stress. **A** The representative fluorescence image of ROS (red) in the liver (*n* = 6). **B** The relative fluorescence intensity of ROS (*n* = 6). **C** The hepatic antioxidant performance indexes (*n* = 6). Data are presented as mean ± SEM. ^*^P < 0.05, ^**^P < 0.01, ^***^P < 0.001

As shown in Fig. 2A, we employed immunofluorescence to assess ROS levels and measured antioxidant enzyme activities. The results revealed that ROS fluorescence intensity was increased after HELP diets and reversed after the addition of 500 and 750 mg/kg CSB (Fig. 2B). Furthermore, in comparison to the CON group, birds fed the HELP diet exhibited a significant decrease in GSH-Px activity and an evident increase in MDA content. (Fig. 2C). However, CH500 and CH750 groups dramatically reversed the GSH-Px and SOD activities, as well as MDA content in line with the result of ROS fluorescence intensity.

### CSB suppressed HELP diet-induced hepatic inflammation

**Fig. 3:**
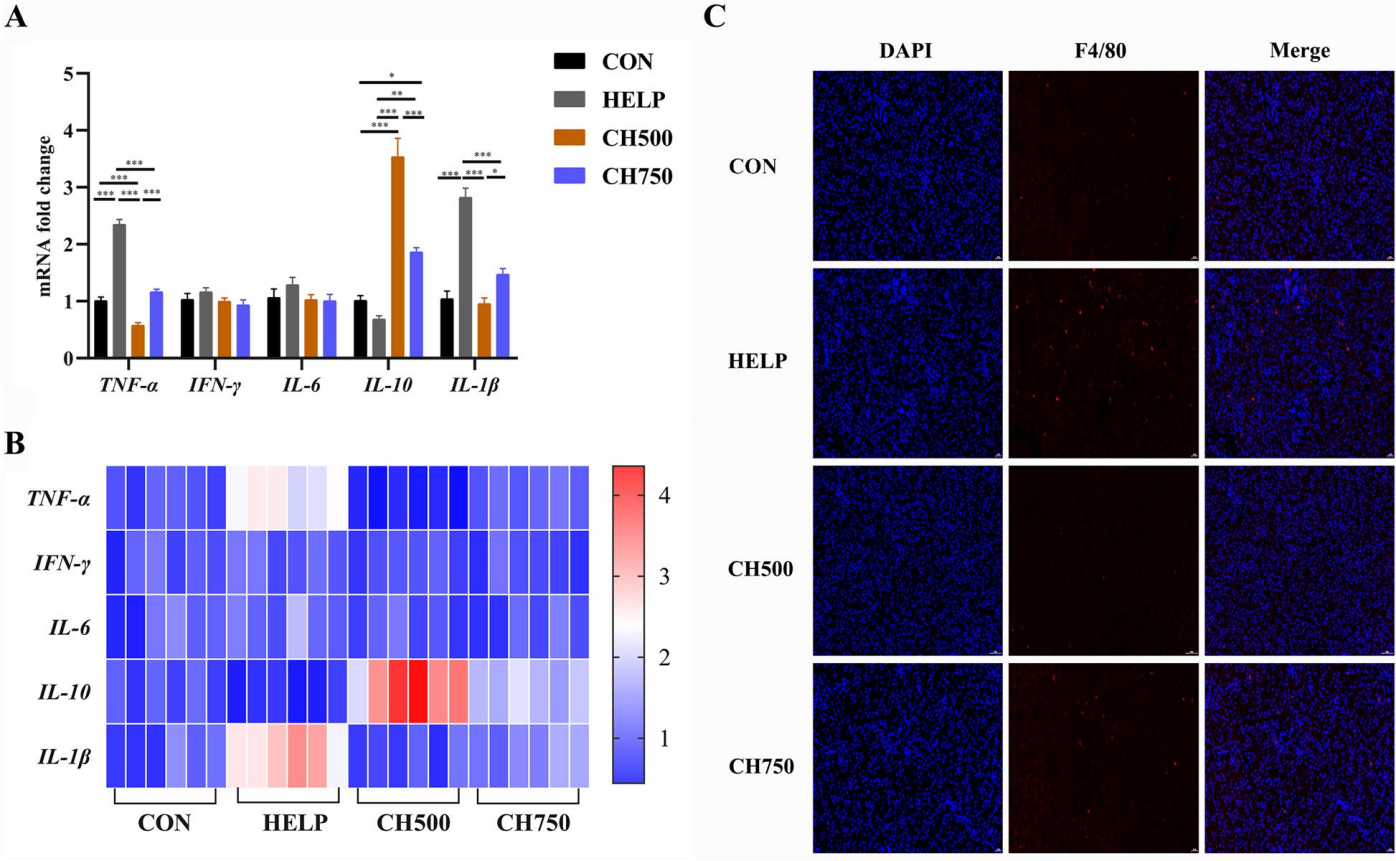
CSB suppressed HELP diet-induced hepatic inflammation. **A** Relative mRNA expression of hepatic inflammatory mediators (*n* = 6). **B** Heat map of relative mRNA gene levels of hepatic inflammatory factors (*n* = 6). **C** Representative immunofluorescence image of F4/80 (red) in the liver (*n* = 6). Data are presented as mean ± SEM. ^*^P < 0.05, ^**^P < 0.01, ^***^P < 0.001

As shown in Fig. 3A, the HELP group markedly upregulated the pro-inflammatory mediators (*TNF-α* and *IL-1β*) mRNA levels and downregulated the anti-inflammatory mediator (*IL-10*) in comparison to the CON group (Fig. 3A and B). Meanwhile, CSB addition significantly reversed these HELP-induced effects. Further inflammation mechanisms were investigated by the detection of hepatic macrophage immunofluorescence. F4/80 immunofluorescence staining showed more macrophages in the HELP administration compared to the CON administration; however, CSB administration efficiently inhibited the macrophage infiltration (Fig. 3C).

### CSB relieved HELP-induced hepatic lipid deposition and metabolic abnormalities

**Fig. 4:**
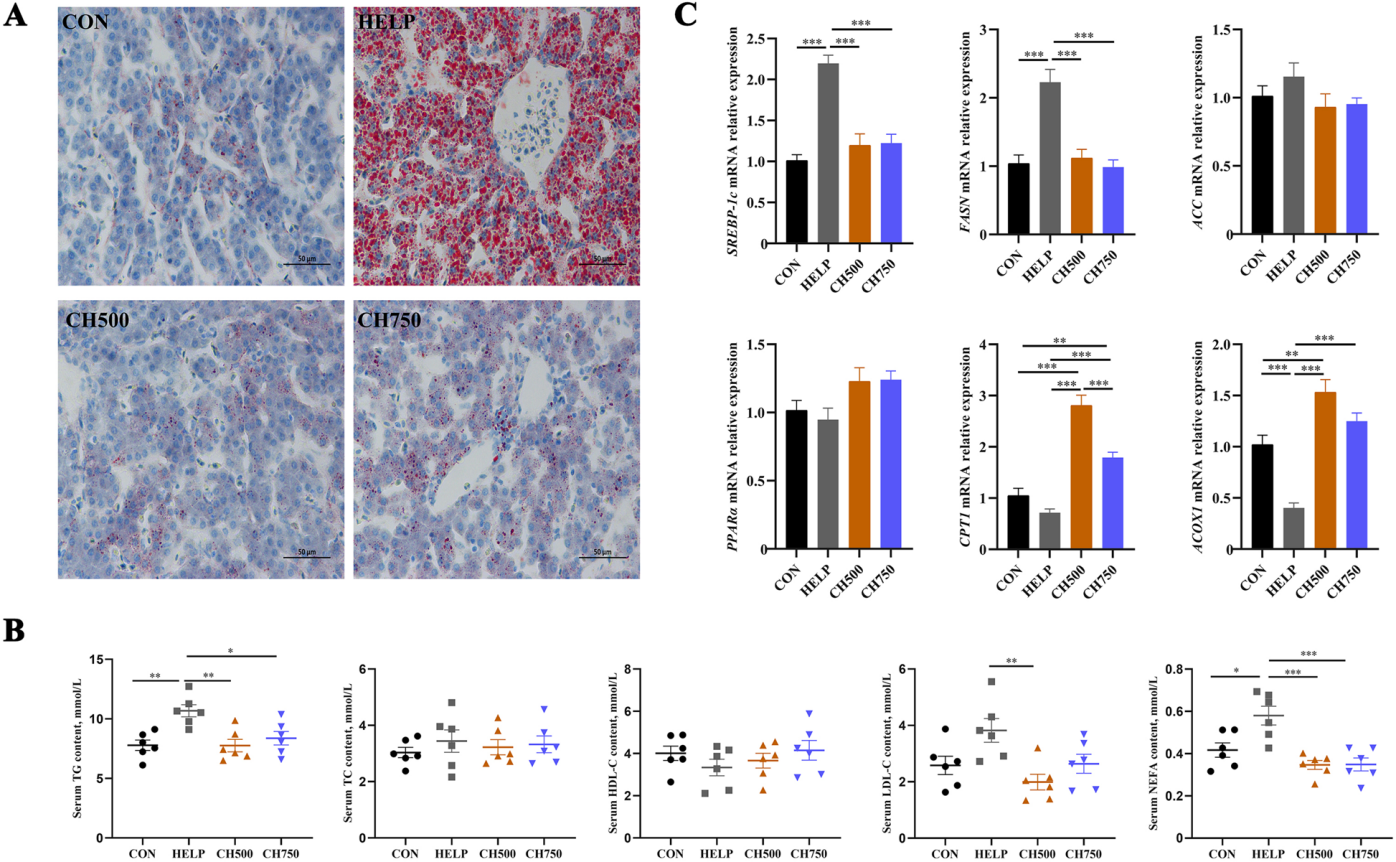
CSB relieved HELP-induced hepatic lipid deposition and metabolic abnormalities. **A** Representative photomicrographs of Oil Red O staining (*n* = 6). **B** Serum lipid profile indexes (*n* = 6). **C** The mRNA levels of lipid metabolism-related genes (*n* = 6). Data are presented as the mean ± SEM. ^*^P < 0.05, ^**^P < 0.01, ^***^P < 0.001

Staining of hepatic tissue sections with Oil Red O revealed that massive lipid droplets occur in HELP diet-fed laying hens (Fig. 4A). Interestingly, the pathological damage induced by the HELP diet reverted to normal levels when supplemented with 500 mg/kg CSB; however, when 750 mg/kg CSB was added, although there was an improvement in pathological damage, it did not completely return to normal levels. Subsequently, we detected the serum lipid profile indexes and observed that the HELP group exhibited elevated serum TG and NEFA levels compared to the CON group, but CH500 or CH750 groups inhibited the rise in TG, LDL-C, and NEFA content caused by HELP diet (Fig. 4B). To confirm the potential effect of CSB on lipid metabolism in HELP-induced laying hens, fatty acid metabolism-related gene expressions were assayed. HELP intake significantly up-regulated the relative transcription level of fatty acid synthesis (*SREBP-1c* and *FASN*), but down-regulated fatty acid oxidation relative transcription levels (*CPT1* and *ACOX1*). Nevertheless, these effects were markedly reversed by CSB treatment (Fig. 4C).

### CSB ameliorated hepatic mitochondrial repair mechanisms disrupted by the HELP diet

**Fig. 5:**
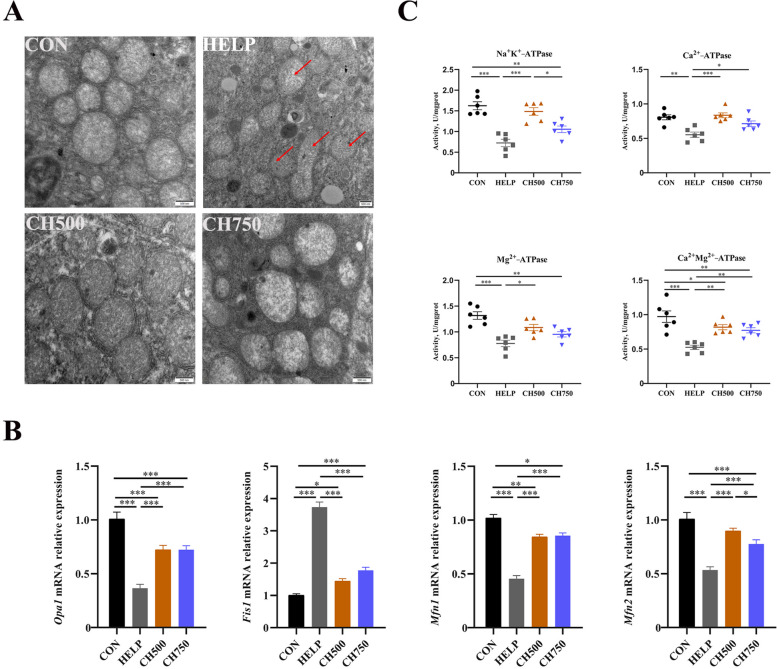
CSB ameliorated hepatic mitochondrial repair mechanisms disrupted by the HELP diet. **A** Representative photomicrographs of mitochondrial morphological changes under TEM (red arrow, damaged mitochondria; *n* = 6). **B** The mRNA levels of mitochondrial dynamic-associated genes (*n* = 6). **C** Changes in the ATPase activity of hepatic mitochondrial (*n* = 6). Data are presented as mean ± SEM. ^*^P < 0.05, ^**^P < 0.01, ^***^P < 0.001

The TEM was used to evaluate the influence of CSB and HELP on the mitochondrial morphological changes of the hepatic tissues. The images revealed that HELP intake induced swollen mitochondria, a significant reduction in mitochondrial cristae, and a cristae breakage phenomenon. However, these adverse effects were reversed by the intake of CSB (Fig. 5A). To thoroughly validate the effect of HELP on mitochondrial dysfunction, we next detected mitochondrial dynamics-related gene expression and ATPase activities (Fig. 5B and C). The results showed that the mRNA levels of *Opa1*, *Mfn1*, and *Mfn2* evidently declined under HELP intake, but they were improved after supplementation with CSB. In addition, the HELP group markedly increased the *Fis1* mRNA expression, and the change was attenuated after administration with CSB. Furthermore, HELP administration significantly decreased the activity of Na^+^K^+^-ATPase, Ca^2+^-ATPase, Mg^2+^-ATPase, and Ca^2+^Mg^2+^-ATPase, whereas their activities were reversed after CSB administration.

### CSB mitigated HELP-induced UPRmt exacerbation

**Fig. 6:**
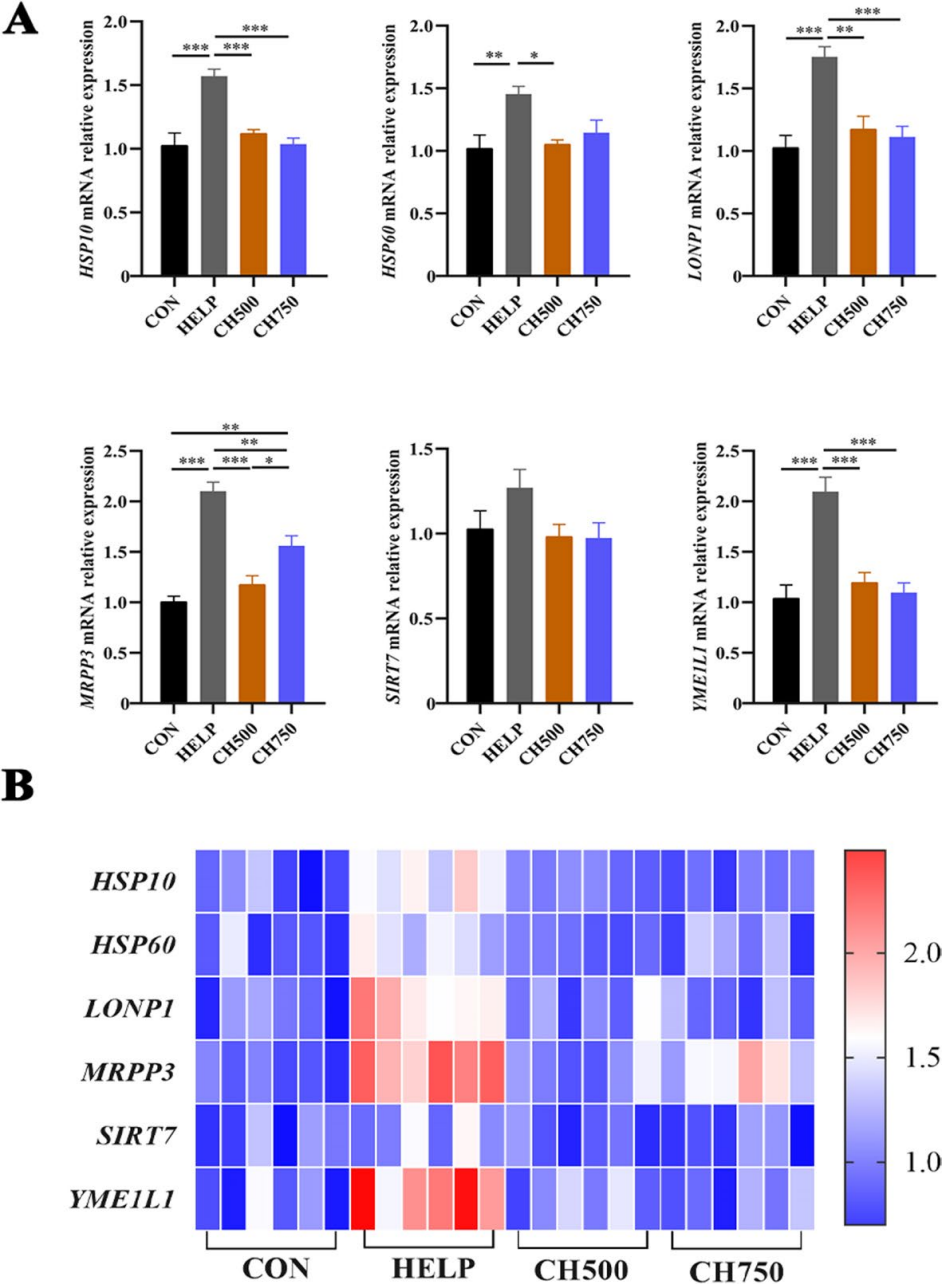
CSB mitigated HELP-induced UPRmt exacerbation. **A** The mRNA-related gene levels of UPRmt (*n* = 6). **B** Heatmap of relative mRNA levels of UPRmt (*n* = 6). Data are presented as mean ± SEM. ^*^P < 0.05, ^**^P < 0.01, ^***^P < 0.001

Mitochondrial denaturation or misfolded proteins accumulation induces UPRmt. We detected the relative mRNA expressions related to UPRmt (Fig. 6A). The results indicated that the HELP diet significantly stimulated the mRNA expression of *HSP10*, *HSP60*, *LONP1*, *MRPP3*, and *YME1L1*, which was reversed by CSB administration. What’s more, the *SIRT7* gene levels were observed with no obvious difference among all groups.

### CSB triggered autophagy in HELP-induced laying hens

**Fig. 7:**
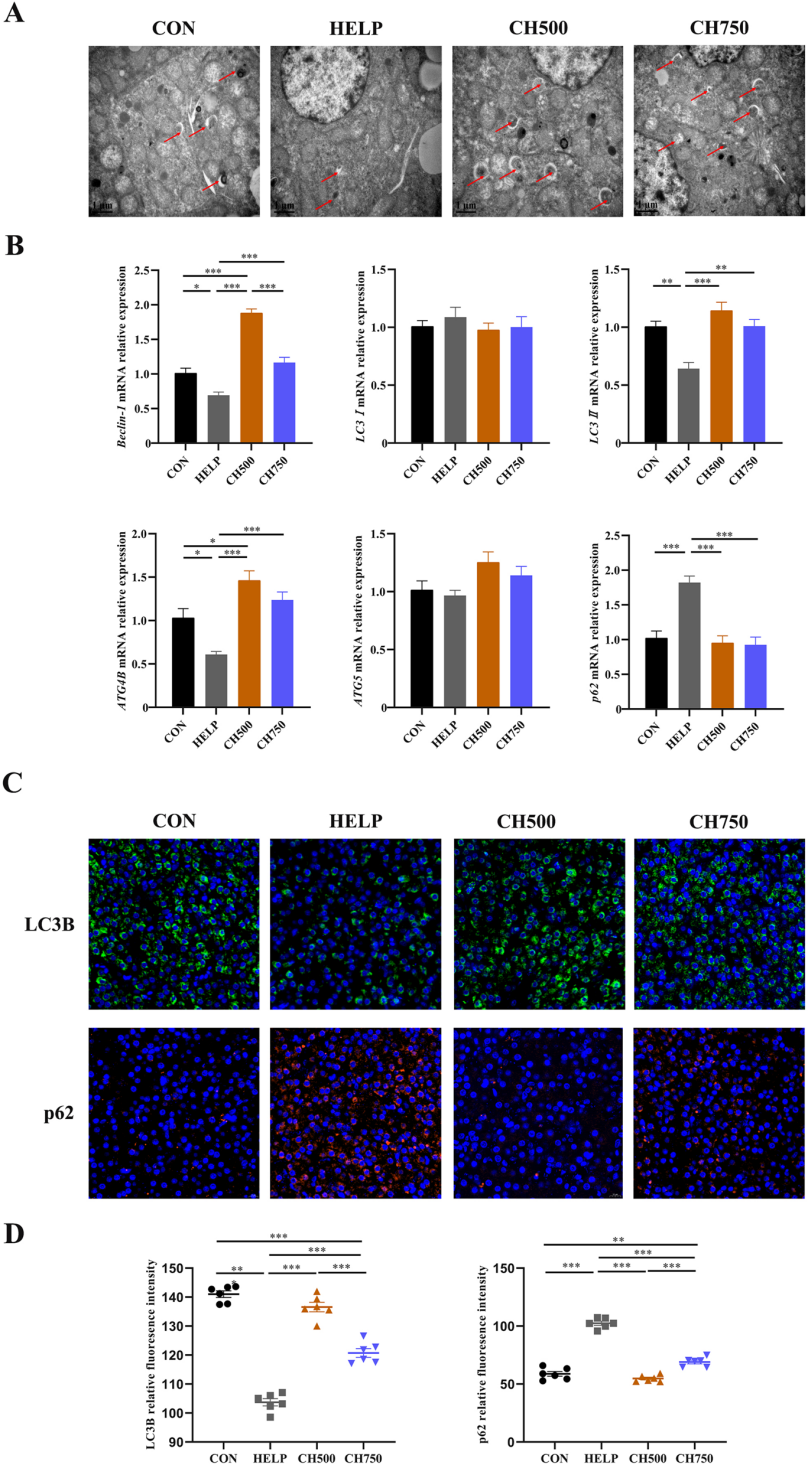
CSB triggered autophagy in HELP-induced laying hens. **A** Representative TEM images showing autophagosome formation (red arrows, autophagosomes; *n* = 6). **B** The mRNA levels of autophagy-related genes (*n* = 6). **C** Representative LC3B (green) and p62 (red) immunofluorescence staining images in hepatic tissue (*n* = 6). Nuclei were stained with DAPI (blue). **D** LC3B and p62 relative fluorescence intensity (*n* = 6). Data are presented as mean ± SEM. ^*^P < 0.05, ^**^P < 0.01, ^***^P < 0.001

Recent studies have emphasized the crucial role of autophagy in regulating lipid metabolism and inflammation. In our study, TEM observation showed a higher number of autophagosomes in the CH500 and CH750 groups when compared to the HELP group (Fig. 7A). Moreover, changes in autophagy-related mediators were assayed. The result illustrated that the CH500 and CH750 groups upregulated the *Beclin-1*, *LC3II*, and *ATG4B* mRNA expressions, but downregulated the level of the *p62* gene expression as compared to the HELP group (Fig. 7B). Finally, we also detected the LC3B and p62 protein expressions relating to autophagy by immunofluorescence (Fig. 7C). CSB triggered HELP-inhibited autophagy, increased LC3B accumulation, and decreased p62 fluorescence intensity (Fig. 7D).

### CSB inhibited apoptosis in HELP-induced laying hens

**Fig. 8:**
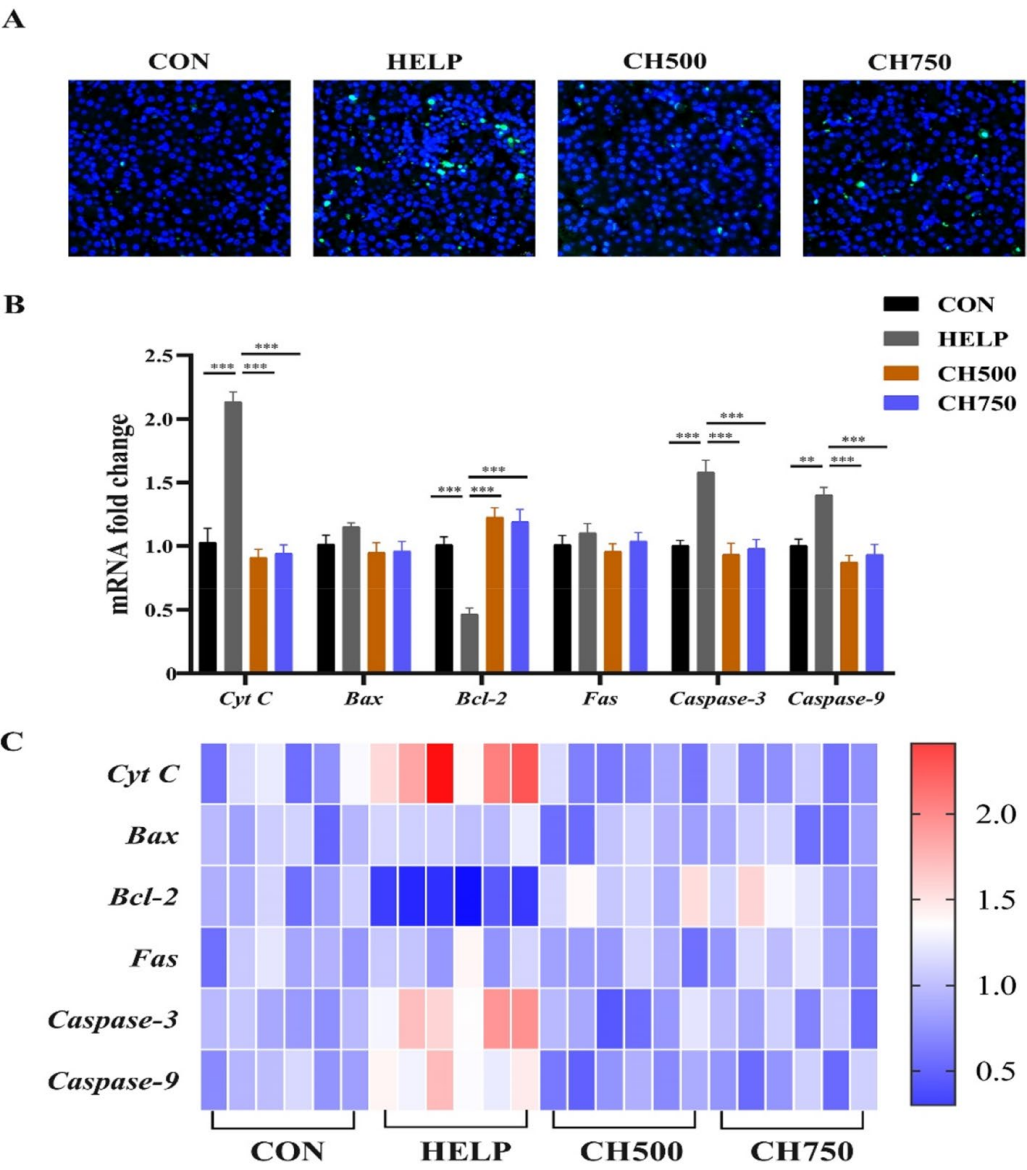
CSB inhibited apoptosis in HELP-induced laying hens. **A** Representative TUNEL (green) immunofluorescence staining images in hepatic tissue (*n* = 6). **B** The mRNA levels of apoptosis-related genes (*n* = 6). **C** Heatmap of mRNA expression of apoptosis-related genes (*n* = 6). Data are presented as mean ± SEM. ^*^P < 0.05, ^**^P < 0.01, ^***^P < 0.001

To verify the effect of CSB and HELP on apoptosis in laying hens, the TUNEL assay was performed. The TUNEL staining result revealed that the green fluorescence intensity was markedly decreased after CSB supplementation in comparison to the HELP group (Fig. 8A). Correspondingly, the HELP group upregulated the gene expression of *Cyt C*, *Caspase-3* and *Caspase-9*, and downregulated the *Bcl-2* level as compared to the CON group; however, their expression was reversed after supplementation with CSB (Fig. 8B and C).

### Correlation analysis and PCA

**Fig. 9:**
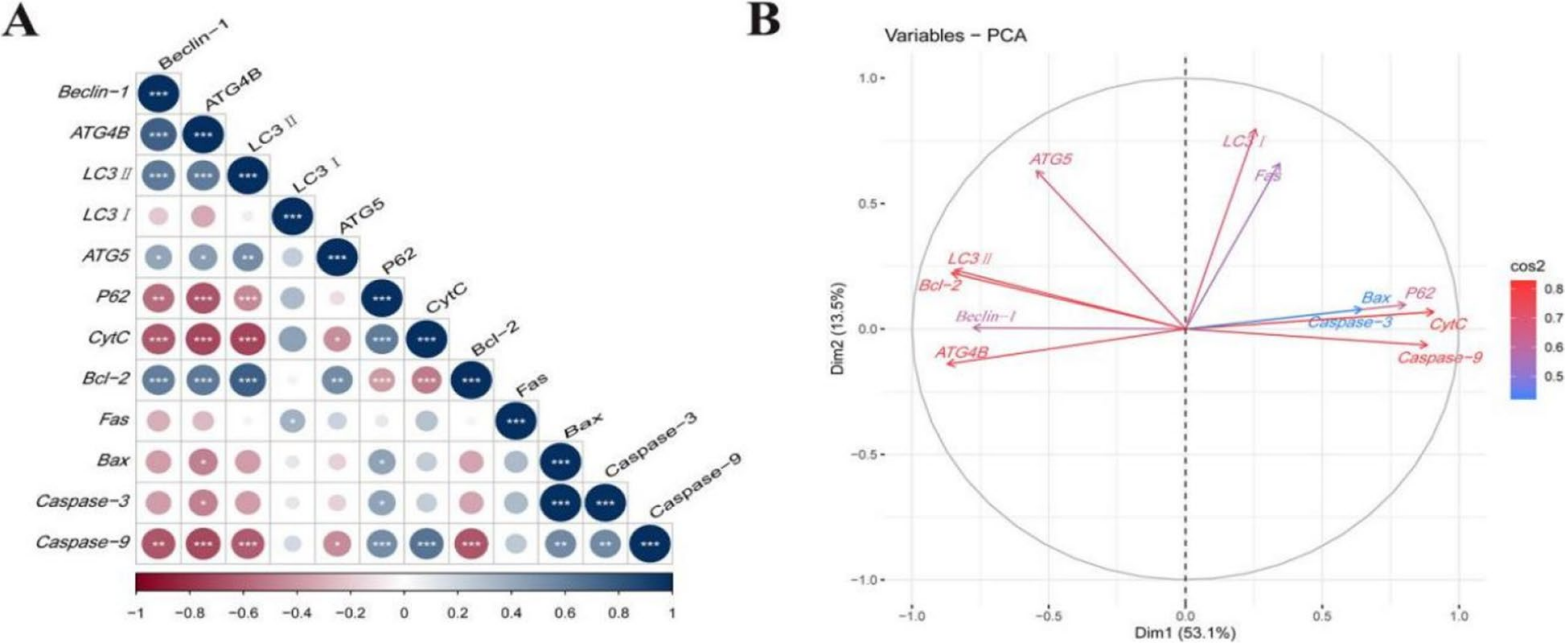
Correlation analysis and PCA. **A** Correlation between autophagy and apoptosis indexes based on Spearman correlation analysis. **B** Principal component analysis of autophagy and apoptosis parameter ranking plots. ^*^P < 0.05, ^**^P < 0.01, ^***^P < 0.001

We then assessed the potential association between autophagy and apoptosis factors using Spearman correlation analysis (Fig. 9A, Table S2 and Fig. S1). The result exhibited that the anti-apoptotic gene *Bcl-2* is positively associated with *ATG5*, *ATG4B*, *Beclin-1*, and *LC3II*. Moreover, the pro-apoptotic genes *CytC*/*Caspase-3*/*Bax*/*Caspase-9* are negatively correlated with *ATG5*, *ATG4B*, *Beclin-1*, and *LC3II*, but positively correlated with *p62*. PCA of autophagy and apoptosis factors is shown in Fig. 9B. Corresponding to the first and second principal components as 53.1% and 13.5%, respectively. The relationships observed in the PCA between autophagy and apoptosis parameters are similar to the above results of Spearman correlation analysis. Moreover, *Bcl-2* had clearly opposite relationships with other apoptosis-related indicators (*Cyt C/Bax/Fas/Caspase-3/Caspase-9*). Similar relationships were observed in *p62* and other autophagy-related indexes (*ATG5*/*ATG4B*/*Beclin-1*/*LC3II*).

## Discussion

In this study, our findings suggest that the HELP diet elicited certain adverse effects on laying hens, which were evaluated through the clinicopathological assessment of the liver. This evaluation revealed the emergence of yellow color, swelling with hemorrhage, and a noticeable elevation in the liver index. In addition, further analysis conducted through HE staining indicated the occurrence of various pathological injuries resulting from the HELP diet, including hepatocyte steatosis, lipid droplet accumulation, and altered hepatocyte shape. These results performed in our research were found to be in line with previous literature [36, 37], which has demonstrated that the HELP diet can trigger the development of FLHS in laying hens. However, we noticed that the typical symptoms of FLHS were ameliorated when CSB was introduced into the diet. Interestingly, we found that the impact of CSB on the liver was not a linear dose–effect relationship, as the liver remained brittle and yellowish-brown at 750 mg/kg, which was less effective than at 500 mg/kg. Plasma enzyme activities like ALT and AST, which are markers of avian FLHS, were used to assess liver damage, as they are released into the bloodstream when hepatocytes are injured [38]. However, we observed that the increase in AST and ALT activities resulting from the HELP diets were notably hindered by the administration of CSB, highlighting the potential of CSB to ameliorate HELP-induced hepatocyte injury.

Dyslipidemia has been widely postulated to be the primary and fundamental engagement in the pathogenesis of NAFLD [39]. Interestingly, the amelioration of dyslipidemia is efficacious in hindering or delaying the advancement of NAFLD [40]. A report by Adeyanju et al. indicated that sodium butyrate supplementation inhibits the progression of dyslipidemia in HFD-induced mice, which was corroborated by the findings of our study that the administration of CSB to HELP-fed laying hens resulted in a remarkable reduction in serum levels of TG, NEFA, and LDL-C [41]. Furthermore, we also revealed that CSB effectively attenuated the massive lipid droplets in hepatic tissues, thus mitigating the progression of FLHS. *SREBP-1c* is regarded as a key orchestrated factor associated with adipogenesis, influencing the mRNA expression related to lipid synthesis, including *FASN* and *ACC* [42]. Existing literature has reported that *SREBP-1c* knockout mice exhibited decreased expression of *ACC1, SCD1,* and *FASN* [43]. Meanwhile, mitochondria and peroxisomes mediate fatty acid oxidation via *CPT1* and *ACOX-1*, respectively. The upregulation of genes encoding *ACOX1* and *CPT1* enhances hepatic fatty acid oxidation, thereby limiting hepatic fat deposition [44]. In line with these findings, our data demonstrated that CSB administration markedly reversed the lipid anabolism-associated gene (*SREBP-1c* and *FASN*) and fatty acid oxidation genes (*CPT1* and *ACOX1*), which were altered anomalously in the HELP group. These findings collectively indicate that the mitigative effect of CSB on HELP-induced FLHS may be ascribed to accelerating fatty acid oxidation and limiting lipid synthesis.

In the broad and deep literature on nutrition and health, it has been well-established that high-fat or high-energy food intake can induce lipid peroxidation in the liver. This, in turn, triggers the accumulation of ROS and diminishes antioxidant capacity, culminating in oxidative damage to liver cells [38, 45]. Butyrate is recognized for its anti-inflammatory and antioxidative characteristics [46]. In this study, a HELP diet evidently elevated ROS fluorescence intensity and MDA content and significantly lowered the activities of GSH-Px and SOD, whereas the addition of CSB reversed it to a relatively normal level, indicating that CSB could effectively ameliorate the oxidative stress in the hepatic tissue. Notably, oxidative stress can trigger inflammatory response pathways, leading to the generation of pro-inflammatory cytokines (*IL-1β*, *IL-1β*, and *TNF-α*) [47], which can further exacerbate the development of NAFLD [48, 49]. For instance, *TNF-α* has been shown to induce hepatocyte necrosis via amplifying ROS in the mitochondria, which triggers lipid peroxidation [50]. Additionally, elevated levels of *IL-1β* have been proved to promote insulin resistance and liver inflammation [51], whereas *IL-10* has the opposite effect [52]. In our study, CSB administration significantly hindered the elevation of pro-inflammatory gene levels (*IL-1β* and *TNF-α*) induced by HELP diets while simultaneously stimulating the secretion of the anti-inflammatory factor *IL-10*, consistent with prior findings [49]. In addition, the CSB group also suppressed the infiltration of inflammatory cells compared with the HELP group, as evidenced by F4/80 immunofluorescence staining, which is in accordance with the changes in inflammatory factors. Overall, the integrated data demonstrate that CSB could validly mitigate liver injury induced by HELP diet via enhancing antioxidant capacity and inhibiting inflammation.

The intricacies of oxidative stress and lipid deposition-induced mitochondrial dysfunction are manifold and complex. The overproduction of lipid deposition and ROS can induce mitochondrial dysfunction, ion balance dysregulation, and membrane abnormalities [53]. Indeed, mitochondria are central sites of ROS generation as well as ATP synthesis [54, 55], which play a crucial role in energy production and oxidative metabolism. Swollen mitochondria and broken ridges were observed by TEM, and ATP supply retardance was shown via decreased ATPase activities, indicating the mitochondrial dysfunction induced by HELP diet. Studies have shown that mitochondrial morphology is formatted under a dynamic balance of fusion and fission events, which have a significant impact on mitochondrial function [56]. Our findings revealed a decrease in mRNA levels of fusion-associated machinery (*Opa1*, *Mfn1*, and *Mfn2*) and an increase in *Fis1* which is correlated with fission machinery, in the HELP group. Nevertheless, the administration of CSB resulted in the reversal of these abnormal changes, manifesting that CSB effectively modulated mitochondrial morphology and alleviated HELP-induced mitochondrial dysfunction. Moreover, the production of ROS and oxidative stress correspond not merely to mitochondrial kinetics but also to intact UPRmt components [57]. Therefore, it is likely that UPRmt is probably another underlying protective mechanism that accounts for the unimpaired mitochondrial function observed in the CSB group. Abundant studies have reported that UPRmt is central to mitochondrial protection and maintenance of mitochondrial homeostasis [58, 59]. When mitochondrial dysfunction occurs, UPRmt is activated and mediates the levels of nuclear-encoded mitochondrial chaperone proteins and proteases, including *HSP10*, *HSP60*, *SIRT7*, *MRPP3*, *LONP*, and *YME1L1*, through transcription factors [60, 61]. To the best of our knowledge, our experiments are the first to evaluate the effect of CSB on UPRmt induced by the HELP diet in laying hens. In the current research, the expressions of *HSP10*, *HSP60*, *LONP1*, *MRPP3*, and *YME1L1* were evidently improved in the HELP group, and the application of CSB prevented this change. Therefore, interestingly, our experiment afforded new evidence that mitochondrial morphology and function were alleviated by CSB through regulating UPRmt and mitochondrial dynamics systems.

Autophagy, a programmed mechanism that orchestrates cellular self-destruction, is employed to recycle cellular components or eliminate damaged organelles [62]. Eliminating malfunctioning or damaged mitochondria through autophagy is vital for maintaining mitochondrial quality [63]. Interestingly, autophagy is also intimately involved in the regulation of cellular lipid balance, and inhibition of autophagy leads to excessive lipid deposition [64]. Emerging evidence has supported that autophagy insufficiency was found in the hepatic tissue of high-fat diet mice [63]. Similarly, in our investigation, we found that the HELP diet led to inhibition of mRNA transcription of critical autophagy genes such as *Beclin-1*, *LC3II*, and *ATG4B*, as well as decreased LC3 protein levels and increased p62 protein levels, which are suggestive of autophagy inhibition. However, the above change was reversed by CSB, and CSB increased the number of autophagosomes. These results imply that CSB may trigger autophagy to prevent fat deposits induced by the HELP diet. Moreover, impaired mitochondria generated more ROS and triggered apoptosis. Existing literature has also demonstrated that autophagy could suppress the activation of apoptosis-associated proteins, facilitating cell survival [65]. A reduction in the *Bcl-2*/*BAX* ratio can result in the release of cytochrome C from the mitochondria to trigger the mitochondrial-dependent cascade, which ultimately leads to cell death [66]. In our trial, anti-apoptotic factor *Bcl-2* was downregulated, and *Caspase-3*, as well as *Caspase-9* were activated in HELP laying hens, whereas these aberrations were reversed by CSB administration. Moreover, accumulated pieces of literature have proved the interplay between autophagy and apoptosis, which mutually adjusts body homeostasis and the fate of cells [45, 67]. Corresponding well with the above evidence, our correlation and PCA analysis results illustrated an antagonistic relationship between autophagy and apoptosis. Consequently, CSB can prevent disruption of lipid metabolism induced by the HELP diet via harmonizing autophagy and apoptosis.

## Conclusions

In conclusion, dietary CSB could ameliorate HELP diet induced hepatic dysfunction by modulating mitochondrial dynamics, autophagy, and apoptosis in laying hens. Consequently, CSB, as a feed additive, exhibited the capacity to prevent FLHS by modulating autophagy and lipid metabolism.

### Supplementary Information

**Additional file 1: Table S1. **Primer used for quantitative real-time PCR fluorescence PCR analysis. **Table S2. **Calculations of the Spearman correlation coefficients of the mRNA expression of autophagy and apoptosis-related genes. **Fig. S1. **Correlation Network between autophagy and apoptosis indexes based on Spearman correlation.

## Data Availability

Data will be made available on request.

## Abbreviations

ALT: Alanine aminotransferase
AST: Aspartate aminotransferase
CAT: Catalase
CSB: Coated sodium butyrate
DHE: Dihydroethidium
FLHS: Fatty liver hemorrhagic syndrome
GSH-Px: Glutathione peroxidase
HDL-C: High-density lipoprotein cholesterol
HELP: High-energy and low-protein diet
H&E: Hematoxylin-eosin
IF: Immunofluorescence
LDL-C: Low-density lipoprotein cholesterol
MDA: Malondialdehyde
NAFLD: Nonalcoholic fatty liver disease。
O.C.T: Optimum cutting temperature
PCA: Principal component analysis
ROS: Reactive oxygen species
SOD: Superoxide dismutase
TC: Total cholesterol
TG: Triglyceride
UPRmt: Mitochondrial unfolded protein response
