# Supplementary material for: Coated sodium butyrate ameliorates high-energy and low-protein diet induced hepatic dysfunction via modulating mitochondrial dynamics, autophagy and apoptosis in laying hens
Source: J Anim Sci Biotechnol. 2024 Feb 2;15:15. doi: 10.1186/s40104-023-00980-8 (PMC10835823; doi:10.1186/s40104-023-00980-8)
Supplement: Supplementary file 1 — Additional file 1: Table S1. Primer used for quantitative real-time PCR fluorescence PCR analysis. Table S2. Calculations of the Spearman correlation coefficients of the mRNA expression of autophagy and apoptosis-related genes. Fig. S1. Correlation Network between autophagy and apoptosis indexes based on Spearman correlation. [file 40104_2023_980_MOESM1_ESM.doc]

**Table S1** Primer used for real-time quantitative fluorescence PCR analysis

| **Target Gene** | **Primer** | **Primer Sequence (5'→3')** | **Accession No.** |  | | | |
| --- | --- | --- | --- | --- | --- | --- | --- |
| *β-Actin* | Forward | TCCCTGGAGAAGAGCTATGAA | NM_205518.1 |  | | | |
|  | Reverse | CAGGACTCCATACCCAAGAAAG |  |  | | | |
| *SREBP-1c* | Forward | GCCATCGAGTACATCCGCTT | NM_204126.2 |  | | | |
|  | Reverse | GGTCCTTGAGGGACTTGCTC |  |  | | | |
| *FASN* | Forward | GAATCCAGAAGGGCCAACGA | NM_205155.4 |  | | | |
|  | Reverse | TCCAAGGGAGCAGCTTTTGT |  |  | | | |
| *ACC* | Forward | TACAGAGGTACCGGAGTGGT | NM_205505.1 |  | | | |
|  | Reverse | TCTTCCCGAAGGGCAAAGAC |  |  | | | |
| *PPARα* | Forward | AGGCCAAGTTGAAAGCAGAA | NM_001001464.1 |  | | | |
|  | Reverse | TTTCCCTGCAAGGATGACTC |  |  | | | |
| *CPT1* | Forward | GGCTCTGGCAGGAGCTACA | XM_040700878.2 |  | | | |
|  | Reverse | CACTGCAGCTGGGATCTTGA |  |  | | | |
| *ACOX1* | Forward | ACTGAGCTGTGTCTCTTGTATG | XM_015295164.2 |  | | | |
|  | Reverse | GCTTCAGGTGTTTGTGGAAAG |  |  | | | |
| *IFN-γ* | Forward | AGCTGACGGTGGACCTATTATT | NM_205149.1 |  | | | |
|  | Reverse | GGCTTTGCGCTGGATTC |  |  | | | |
| *TNF-α* | Forward | GACAGCCTATGCCAACAAGTA | AY765397.1 |  | | | |
|  | Reverse | TCCACATCTTTCAGAGCATCAA |  |  | | | |
| *IL-6* | Forward | CTCGTCCGGAACAACCTCAA | NM_204628.2 |  | | | |
|  | Reverse | TCAGGCATTTCTCCTCGTCG |  |  | | | |
| *IL-10* | Forward | CCAGGGACGATGAACTTAACA | NM_001004414.2 |  | | | |
|  | Reverse | GATGGCTTTGCTCCTCTTCT |  |  | | | |
| *IL-1β* | Forward | ACTGGGCATCAAGGGCTA | XM_015297469.2 |  | | | |
|  | Reverse | GGTAGAAGATGAAGCGGGTC |  |  | | | |
| *FIS1* | Forward | GGACGACCTGCTGAAGTTTG | XM_006773401.2 |  | | | |
|  | Reverse | CGTTGTACTTGCTTCGCACC |  |  | | | |
| *Mfn1* | Forward | AGTTCTGCCGGACACTGAAG | XM_015291425.4 |  | | | |
|  | Reverse | CCTCCGCCATGATGTAACGAA |  |  | | | |
| *Mfn2* | Forward | ACTTCCATGGGGATCATCGT | XM_015882356.1 |  | | | |
|  | Reverse | GGCGTTCATACACATAAAGCAG |  |  | | | |
| *Opa1* | Forward | TGCACTGTTAGCACT | XM_015871821.1 |  | | | |
|  | Reverse | CTCCAGGTAAATCGACCA |  |  | | | |
| *HSP10* | Forward | CCCCTGTTTGATCGTGTT | NM_205067.3 |  | | | |
|  | Reverse | TCCCTTGAGCTTTTTCTG |  |  |  |  |  |
| *HSP60* | Forward | CTACAGGTGGTGCTGTGT | NM_001012916.3 |  | | | |
|  | Reverse | ATGGTGTCATCTTTGGTC |  |  | | | |
| *LONP1* | Forward | CTGGAGCAAAGGAGCAAG | XM_001232111.7 |  | | | |
|  | Reverse | CAAAGGGTTCAAGGCAAT |  |  | | | |
| *MRPP3* | Forward | CCGTCAACTGTTTGCTGT | XM_421241 |  | | | |
|  | Reverse | ATCCTCATCCCATCCTTT |  |  | | | |
| *SIRT7* | Forward | GGGAAGCAGCAACAGAAGC | NM_001291971.1 |  | | | |
|  | Reverse | TTTGGACGACGAGGTGGG |  |  | | | |
| *YME1L1* | Forward | CACAACAGGTGCTTCCAG | NM_001031512 |  | | | |
|  | Reverse | TCGTATGAGTCCCGTAGA |  |  | | | |
| *Bec*lin-1 | Forward | CGACTGGAGCAGGAAGAAG | NM001006332 |  | | | |
|  | Reverse | TCTGAGCATAACGCATCTGG |  |  | | | |
| *LC3-I* | Forward | TTACACCCATATCAGATTCTTG | XM417327 |  | | | |
|  | Reverse | ATTCCAACCTGTCCCTCA |  |  | | | |
| *LC3-II* | Forward | CTTCTTCCTCCTGGTGAACG | NM001031461 |  | | | |
|  | Reverse | GCACTCCGAAAGTCTCCTGA |  |  | | | |
| *ATG4B* | Forward | AGGTGGATAAAAGGCAAGAGG | NM213573 |  | | | |
|  | Reverse | GTCCATACCACTGACCGATG |  |  | | | |
| *ATG5* | Forward | GGCACCGACCGATTTAGT | NM001006409 |  | | | |
|  | Reverse | GCTGATGGGTTTGCTTTT |  |  | | | |
| *p62* | Forward | TGCTGGAGGTATTGAAGT | XP_004945011.1 |  | | | |
|  | Reverse | CAGTATGGTTTGGTCTGA |  |  | | | |
| *Cyt c* | Forward | GCCAAACATCCAAACACAGA | NM001079478 |  | | | |
|  | Reverse | AGGCAAGCACAAGACTGGA |  |  | | | |
| *Bax* | Forward | GTGGTCAGTCCGAGCCTTTT | XM001235092 |  | | | |
|  | Reverse | TCCATTCAGGTTCTCTTGACC |  |  | | | |
| *Bcl-2* | Forward | ATCGTCGCCTTCTTCGAGTT | NM205339 |  | | | |
|  | Reverse | ATCCCATCCTCCGTTGTTCT |  |  | | | |

### *SREBP-1c*, [sterol regulatory element binding transcription factor 1](https://www.ncbi.nlm.nih.gov/gene/78968)c; *FASN*, fatty acid synthase; *ACC*, acetyl-CoA carboxylase alpha; *PPARα*, peroxisome proliferator activated receptor alpha; *CPT1*, carnitine palmitoyltransferase 1; *ACOX1*, acyl-CoA oxidase 1; *IFN-γ*, interferon gamma; *TNF-α*, tumour necrosis factor alpha; *IL-6*, interleukin 6; *IL-10*, interleukin 10; *IL-1β*, interleukin 1, beta; *FIS1*, fission, mitochondrial 1; Mfn1, mitofusin 1; *Mfn2*, mitofusin 2; *Opa1*, optic atrophy 1; *HSP10*, heat shock protein 10; *HSP60*, heat shock protein 60; *LONP1*, lon peptidase 1; *MRPP3*, mitochondrial ribonuclease P protein 3; *SIRT7*, sirtuin 7; *YME1L1*, YME1 like 1 ATPase; *LC3-I*, microtubule associated protein 1 light chain 3 alpha; *LC3-II*, microtubule associated protein 1 light chain 3 beta; *ATG4B*, autophagy related 4B cysteine peptidase; *ATG5*, autophagy related protein 5; *p62*, sequestosome 1; *Cyt c*, cytochrome c; *Bax*, BCL2-associated X protein; *Bcl-2*, B-cell lymphoma-2

**Table S****2** Calculations of the Spearman correlation coefficients of the mRNA expression of autophagy and apoptosis-related genes

| **Gene** | **Beclin1** | **ATG4B** | **LC3Ⅱ** | **LC3Ⅰ** | **ATG5** | **p62** | **CytC** | **Bcl2** | **Fas** | **Bax** | **Caspase3** | **Caspase9** |
| --- | --- | --- | --- | --- | --- | --- | --- | --- | --- | --- | --- | --- |
| Beclin-1 | 1.000 |  |  |  |  |  |  |  |  |  |  |  |
| ATG4B | 0.758 | 1.000 |  |  |  |  |  |  |  |  |  |  |
| LC3B | 0.635 | 0.630 | 1.000 |  |  |  |  |  |  |  |  |  |
| LC3A | -0.208 | -0.327 | -0.065 | 1.000 |  |  |  |  |  |  |  |  |
| ATG5 | 0.421 | 0.466 | 0.545 | 0.238 | 1.000 |  |  |  |  |  |  |  |
| p62 | -0.568 | -0.669 | -0.452 | 0.329 | -0.126 | 1.000 |  |  |  |  |  |  |
| CytC | -0.645 | -0.714 | -0.730 | 0.446 | -0.432 | 0.622 | 1.000 |  |  |  |  |  |
| Bcl-2 | 0.616 | 0.637 | 0.770 | 0.048 | 0.548 | -0.387 | -0.510 | 1.000 |  |  |  |  |
| Fas | -0.309 | -0.263 | -0.045 | 0.364 | 0.200 | 0.095 | 0.282 | -0.043 | 1.000 |  |  |  |
| Bax | -0.370 | -0.485 | -0.374 | 0.103 | -0.177 | 0.431 | 0.239 | -0.342 | 0.325 | 1.000 |  |  |
| Caspase3 | -0.370 | -0.485 | -0.374 | 0.103 | -0.177 | 0.431 | 0.239 | -0.342 | 0.325 | 1.000 | 1.000 |  |
| Caspase9 | -0.650 | -0.701 | -0.635 | 0.170 | -0.449 | 0.541 | 0.670 | -0.657 | 0.256 | 0.550 | 0.550 | 1.000 |


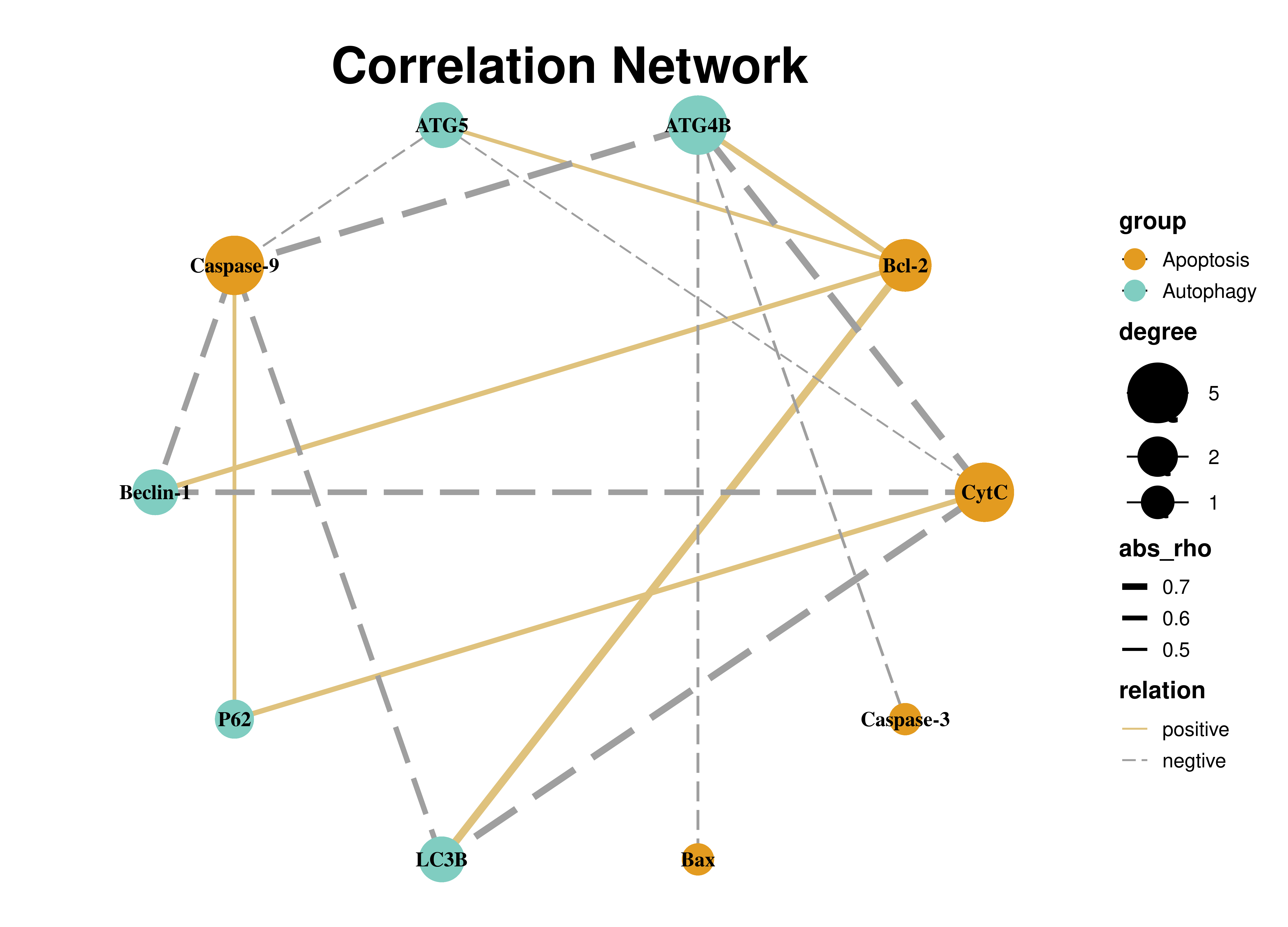


**Fig. S1** Correlation Network between autophagy and apoptosis indexes based on Spearman correlation
